# P-817. Comparison of swab versus fluid culture obtained from infected sites in the operating room

**DOI:** 10.1093/ofid/ofaf695.1025

**Published:** 2026-01-11

**Authors:** Monica Lou, Avvi R Shabat, Priya Amin, Raymond Lopez, Kenneth Muldrew, Daniel M Musher

**Affiliations:** Baylor College of Medicine, Houston, TX; MEDVAMC Houston, TX, Houston, Texas; Michael E. DeBakey VA Medical Center, Houston, Texas; Baylor College of Medicine, Houston, TX; Baylor College of Medicine, Houston, TX; Michael E. DeBakey VA Medical Center / Baylor College of Medicine, Houston, Texas

## Abstract

**Background:**

Surgically obtained specimens from clinically infected sites are regularly submitted from the operating room for Gram stain and culture, with results used to inform empiric and, then, definitive antibiotic therapy. Guidelines on utilization of the microbiology laboratory state that fluid or tissue specimens are strongly preferred to swab. We are not aware of a prospective study that examines this recommendation.

**Methods:**

In this ‘real-world’ study, surgeons were asked at surgery to submit for Gram stain and culture both a swab with gel transport medium and infected fluid in a sterile syringe or tissue from the same infected site. We evaluated concordance between Gram stain and culture for each individual specimen and then between swab vs the fluid or tissue specimens. Results were regarded as concordant if the same predominant organism(s) were identified by Gram stain and by culture. Organisms generally regarded as non-pathogens and/or reported as ‘rare growth’ were excluded from the analysis.

**Results:**

A total of 71 pairs of specimens were collected from 68 patients who ranged in age from 28 to 92 years old. Sixty-three (93%) were male. Samples obtained were from various anatomical sites, predominantly from a lower extremity.

For 39 of 71 (54.9%) swab samples, Gram stain and culture showed the same predominant organism(s) (concordance), compared to 53 of 71 (74.6%) samples of fluid or tissue (P = 0.03). Of the 17 cases in which results of culture of fluid or tissue and swab were not concordant, in 11 (64.7%) a bacterial pathogen was isolated from fluid or tissue but not from swab culture. In 4 (23.5%) cases, the swab culture yielded pathogenic bacteria that were not identified in fluid or tissue (11 of 17 vs 4 of 17, P = 0.04). In the 2 remaining cases, different pathogenic bacteria were isolated from cultures of fluid and swab.

**Conclusion:**

Our study shows the validity of Gram stain and provides data to support current guidelines by documenting that Gram stain and culture of infected fluid or tissue yield more relevant data than Gram stain and culture of swab specimens. However, multiple specimens may provide even more reliable results. By enabling more directed selection of antibiotics, greater use of Gram stain and culture of liquid and tissue specimens may play an important role in antibiotic stewardship.

**Disclosures:**

All Authors: No reported disclosures

